# The feedback intervention trial: a national stepped wedge cluster randomised controlled trial to improve hand hygiene

**DOI:** 10.1186/1753-6561-5-S6-O66

**Published:** 2011-06-29

**Authors:** S Stone, the NOSEC-FIT group

**Affiliations:** 1 Universitry College London, London, UK; 2 Hand Hygeine Liason Group, London, UK

## Introduction / objectives

Achieving a sustained improvement in hand hygiene compliance is WHO’s first global patient safety challenge. There is no RCT evidence showing how to do this. Systematic reviews require long term well designed RCT be done, testing effectiveness of a behavioural intervention designed using behavioural theory.

## Methods

Three year stepped wedge cluster RCT of a feedback intervention in 16 English/Welsh Hospitals (16 ITUs; 44 Acute Care of the Elderly [ACE] wards, routinely implementing a national cleanyourhands campaign), testing null hypothesis that intervention no more effective than routine practice.

Intervention-based on Goal & Control theories. Repeating 4 week cycle of 20 mins observation, feedback & personalised action planning, recorded on forms. Computer generated stepwise randomisation.

Primary outcome: direct blinded observation of hand hygiene compliance (%).

## Results

All 60 wards randomised, 33 implemented intervention (11 ITU 22 ACE). Mixed effects regression analysis (all wards) accounting for confounders, temporal trends, ward type & fidelity to intervention (forms/month used).

Intention to treat (ITT) analysis: estimated odds ratio (OR) for hand hygiene compliance rose post-randomisation (1.44; 95% CI 1.18, 1.76;p<0.001) in ITUs but not ACE wards, equivalent to 7-9% absolute increase in compliance.

Per protocol analysis for implementing wards: OR for compliance rose for both ACE (1.67 [1.28-2.22];p<0.001) & ITUs (2.09 [1.55-2.81];p<0.001) equating to absolute increases of 10-13% & 13-18% respectively. OR fell or unchanged on non-implementing ACE & ITU wards. Fidelity to intervention closely related to compliance on ITUs (OR 1.12 [1.04, 1.20];p=0.003 per completed form).

## Conclusion

Despite difficulties in implementation, ITT, per protocol & fidelity to intervention analyses showed an intervention coupling feedback to personalised action planning significantly improved hand hygiene compliance, in wards implementing a national handhygiene intervention. Further implementation studies are needed to maximise the intervention’s effect in different settings.

## Disclosure of interest

S. Stone Grant/Research support from GOJO.

